# Analysis of the ASMT Gene Family in Pepper (*Capsicum annuum* L.): Identification, Phylogeny, and Expression Profiles

**DOI:** 10.1155/2019/7241096

**Published:** 2019-04-02

**Authors:** Luzhao Pan, Jiaqiu Zheng, Jia Liu, Jun Guo, Fawan Liu, Lecheng Liu, Hongjian Wan

**Affiliations:** ^1^College of Horticulture and Gardening, Yangtze University, Jingzhou 434025, China; ^2^State Key Laboratory Breeding Base for Zhejiang Sustainable Pest and Disease Control, Institute of Vegetables, Zhejiang Academy of Agricultural Sciences, Hangzhou 310021, China; ^3^Jiangsu Coastal Area Institute of Agricultural Sciences, Yancheng 224002, China; ^4^Wulanchabu Academy of Agricultural Sciences, Wulanchabu 012000, China; ^5^Horticultural Research Institute, Yunnan Academy of Agricultural Science, Kunming 650231, China

## Abstract

Acetylserotonin methyltransferase (ASMT) in plant species, one of the most important enzymes in melatonin biosynthesis, plays a rate-limiting role in the melatonin production. In this study, based on the whole genome sequence, we performed a systematic analysis for the ASMT gene family in pepper (*Capsicum annuum* L.) and analyzed their expression profiles during growth and development, as well as abiotic stresses. The results showed that at least 16 CaASMT genes were identified in the pepper genome. Phylogenetic analyses of all the CaASMTs were divided into three groups (group I, group II, and group III) with a high bootstrap value. Through the online MEME tool, six distinct motifs (motif 1 to motif 6) were identified. Chromosome location found that most CaASMT genes were mapped in the distal ends of the pepper chromosomes. In addition, RNA-seq analysis revealed that, during the vegetative and reproductive development, the difference in abundance and distinct expression patterns of these CaASMT genes suggests different functions. The qRT-PCR analysis showed that high abundance of CaASMT03, CaASMT04, and CaASMT06 occurred in mature green fruit and mature red fruit. Finally, using RNA-seq and qRT-PCR technology, we also found that several CaASMT genes were induced under abiotic stress conditions. The results will not only contribute to elucidate the evolutionary relationship of ASMT genes but also ascertain the biological function in pepper plant response to abiotic stresses.

## 1. Introduction

Melatonin (N-acetyl-5-methoxytryptamine) was found firstly in 1958 by Aaron B. Lerner and ubiquitously exists in kinds of taxonomically distant groups of organisms, such as algae, bacteria, protozoa, fungi, and plants [1–3]. It is well known that melatonin is considered as critical bioactive molecule affecting the growth of plants and animals [4]. Previous researches had reported that it was present in all plant species studied and was found in various tissues of higher plants, including roots, stems, leaves, flowers, fruits, and seeds [5–7]. In fact, melatonin plays an active role in the plant growth and development stages, which can be used as protective agent to prevent oxidative damage resulting from exposure to heavy metals, cold, high temperatures, salt, and peroxidizing chemicals [8–10]. However, the potential function about melatonin in plants, thus far, is still insufficient.

In 1995, Dubbels et al. reported that melatonin existed in various plant species. However, due to a form of unstable indoleamine or extremely low levels of expression in plants, the biosynthetic pathways and physiological roles of melatonin are in their infancy [11, 12]. Fortunately, based on recent reports, four consecutive enzymes involved in biosynthesis of melatonin, which include tryptophan decarboxylase (TDC), tryptamine 5-hydroxylase (T5H), serotonin N-acetyltransferase (SNAT), and N-acetylserotonin methyltransferase (ASMT), were verified through the *in vivo* analysis [13, 14]. Among these four members, a single copy T5H [15] and SNAT gene [16] encode the respective enzyme in rice, while each of TDC [17] and ASMT [18] was constituted by a small gene family in rice.

Recently, the researchers found that two of these four enzymes (SNAT and ASMT) were very critical for regulating the relative melatonin levels in all studied plants, since their catalytic activities are very low compared to the other two genes [19–23]. In addition, in 2014, Kang et al. proposed that rice ASMT mRNA can be induced by various biotic and abiotic stresses, such as abscisic acid (ABA), acifluorfen, salt, and copper treatments [18]. Subsequently, some studies have shown that overexpression of ASMT1, ASMT2, and ASMT3 enhanced the enzyme activity in rice. Interestingly, two of the rice genes (ASMT1 and ASMT2) were highly expressed in the stems and flowers, but the transcript of ASMT3 was barely observed in all of the tested tissues. Moreover, these three ASMTs of mRNAs can be synchronously induced in the treatments with abscisic and methyl jasmonic acids [19].

Pepper (*Capsicum annuum* L.), an important vegetable crop in the world, is deeply loved by wide parts of the population since it is a major ingredient in cuisines, essential vitamins, and other healthy nutrients [24]. Nowadays, it is a great task to study the reproductive development and increase the yield and fruit quality of pepper crops. Coincidentally, melatonin, a novel plant growth regulator, was used as modulator of reproductive development, control of root and shoot organogenesis, and maintenance of plant tissues [25]. In the present paper, to comprehensively decipher the function of melatonin in the pepper plants, a systematic analysis of ASMT genes was performed using the bioinformatics method. Here, our aim was to (i) identify the members of CaASMT gene family, (ii) analyze the phylogenic relationship of CaASMT genes, and (iii) reveal their expression patterns in various tissues by using RNA-seq. In conclusion, our results will provide an insight into the evolutionary patterns of the CaASMTs in pepper, as well as contribute to laying a foundation for understanding the function of CaASMTs in the future.

## 2. Materials and Methods

### 2.1. Genome-Wide Identification of CaASMT Family Members in Pepper

To identify the CaASMT gene family members, the predicted pepper gene sequences were downloaded from the Pepper Genome Database (PGD, http://peppergenome.snu.ac.kr/) [26]. Then, a local database was constructed using BioEdit 7.0 software [27]. BlastP searches were performed using the conserved domain of rice ASMT protein (AK069308) as a query sequence in local database. The pepper ASMT genes with e-value less than 1e-5 were used for further analysis. Then, the Hidden Markov Model (HMM) profile of the O-methyltransferase (PF00891.17) was used to retrieve against the local database with e-value <1e-5. Finally, all of the putative CaASMT sequences with incomplete domain were excluded by HMM analysis.

The physicochemical properties of CaASMT genes were analyzed using the online ExPASy-ProtParam tool (http://web.expasy.org/protparam/) [28], which included a number of amino acids, molecular weight (MW), theoretical isoelectric point (pI), and instability index (II; with the value >40 pass for unstable) of deduced CaASMT proteins.

### 2.2. Phylogenetic Relationship and Conserved Motifs

For the purpose of probing evolutionary relationships among CaASMT genes in the pepper, the full-length protein sequences were aligned using ClustalX with default settings [29]. An unrooted phylogenetic tree was constructed using the MEGA7.0 software based on the above results [30]. The parameters for constructing a phylogenetic tree are as follows: the statistical method of maximum likelihood, bootstrap replication 1000 times, uniform rates, and pairwise deletion.

Conserved motifs were identified using the online tools Multiple Em for Motif Elicitation (MEME Suite 4.12.0) (http://meme-suite.org/tools/meme) program [31]. The optimized parameters of MEME included the following: the maximum number of each genes was 8, the smallest and largest width of each motif were 6 and 50 amino acid sequences, respectively, and the remaining parameters are set by default settings.

### 2.3. Chromosomal Location Analysis

The information about the CaASMT gene location was extracted from the website of pepper sequence genomics (http://peppersequence.genomics.cn/page/species/mapview.jsp). Chromosome mapping of the CaASMT genes was determined via the software MapDraw2.1 [32]. Subsequently, tandem duplication and segmental duplication were also further expounded. The tandemly duplicated genes were identified with two criteria: two or more CaASMT genes within a range of 100 kb distance; the similarity is no less than 70% [33–35]. Besides, the online tool Plant Genome Duplication Database (PGDD, http://chibba.agtec.uga.edu/duplication/index/locus) was applied to investigate the segmental duplication genes.

### 2.4. Expression Profiles of CaASMT Genes Based on RNA-Seq

To investigate the expression profiles of the CaASMT gene family, the expression levels of these genes in the growth and development of pepper, as well as under abiotic stress treatments, were analyzed. To analyze the expression patterns of the CaASMT members among different developmental stages of organs, the RNA-seq atlas was downloaded from the PGD website (http://peppersequence.genomics.cn/page/species/index.jsp). Five different organs, including leaf, flower, placenta, seed, and pericarp, were selected for expression analysis.

Subsequently, Reads Per Kilo bases per Million mapped Reads (RPKM) expression values of CaASMT genes were log2 transformed [36]. The hierarchically clustering was performed according to Pearson's correlation distance with average linkage, and gene clusters were classified by expression values. Heat maps (including RNA-seq and abiotic stress treatment) representing digital gene expression analysis were generated using the Multi-Experiment Viewer (MeV) [37] according to the above steps.

### 2.5. Plant Materials, Heat Stress Treatment, and RT-qPCR Analysis

A cultivated pepper (*C. annuum* L.), grown at Zhejiang Academy of Agricultural Sciences, was used in this experiment. Fruit tissues from four different developmental stages were collected: small fruit, mature green fruit, breaker fruit, and mature red fruit. Heat treatment was performed at young plants; plants were first grown at 23°C under normal conditions and then transferred to 42°C. Treated plant leaves were collected at 0, 3, 6, and 12 h posttreatment. Three biological duplications were performed. Subsequently, all the samples collected were immediately frozen in liquid nitrogen and stored at -80°C.

Total RNAs were extracted from each tissue using the RNA simple Total RNA Kit (TIANGEN, Beijing, China) and then were reverse transcribed into cDNA using the FastQuant RT Kit with gDNase (TIANGEN, Beijing, China) according to the manufacturer's instructions. The first-strand cDNA was synthesized using PrimeScript^TM^ RT Reagent Kit (TaKaRa, Dalian, China) and was further diluted 1 : 9 with nuclease-free water. Gene-specific primers of the CaASMT genes were designed using the primer 5.0 software (Supplemental Table S1).

The RT-qPCR reactions were performed in a volume of 20 *μ*l : 7.8 *μ*l of distilled water, 10 *μ*l of Super Mix, 0.6 *μ*l of each primer (forward and reverse), 2 *μ*l Rox Dye, and 1 *μ*l diluted cDNA from the samples. The PCR was conducted by the following procedure: 95°C for 30 s, followed by 40 cycles at 95°C for 5 s, 55°C for 15 s, and 72°C for 10 s. The quality and specificity of each primer were determined by the melt curve [38]. The UBI gene was used as an internal reference for further expression analysis [39]. Three independent replicates were then performed for each of the CaASMT genes. The value of relative expression was analyzed using the 2^-△△Ct^ method.

## 3. Results

### 3.1. Genome-Wide Identification of the CaASMT Gene Family in Pepper

A total of 33 candidates were initially gotten using the HMM search program, seventeen of which were manually removed since their homologs are less than 31% amino acid identities compared with ASMTs in rice [40, 41]. In total, 16 nonredundant CaASMT genes were identified (Table 1). As a matter of convenience, we proposed an acronym of nomenclature that the pepper ASMTs can be named CaASMT01 to CaASMT16.

The detailed information of each identified CaASMT, including gene name, locus name, chromosome location, protein length, molecular weight (MW), isoelectric point (pI), and instability index, was presented in Table 1. Molecular analysis indicated that the length of predicted protein sequences of these pepper CaASMT genes varied from 238 (CaASMT04) to 372 residues (CaASMT15). Accordingly, MW varied from 26.22 (CaASMT04) to 42.04 kDa (CaASMT15), whereas the predicted pI-values of these genes ranged from 4.75 (CaASMT04) to 6.22 (CaASMT11). The pI-values indicated that all of CaASMT proteins were acidic (pI-values < 7). Besides, the instability index showed that 13 CaASMT genes (the value < 40) was deemed to be stable proteins, and only 3 genes (CaASMT11, 01, and 16) with the value > 40 were unstable proteins.

### 3.2. Phylogenetic Analysis and Conserved Motifs

**Figure 1:**
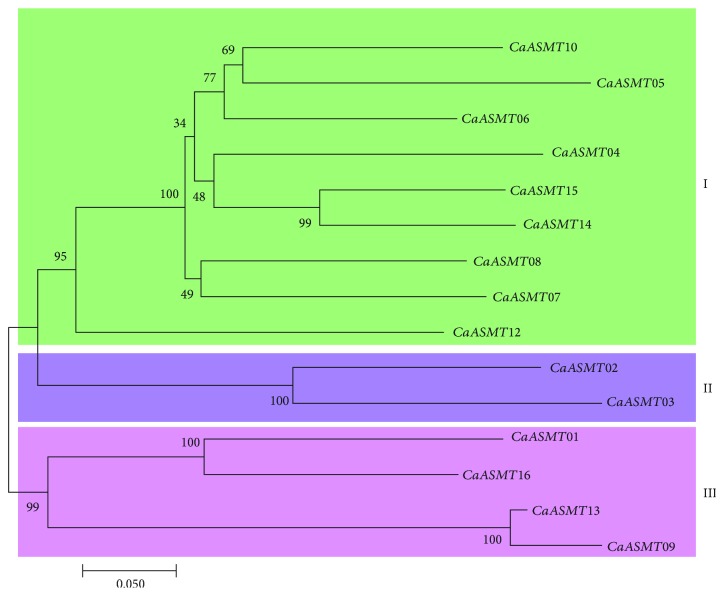
Phylogenetic analysis of the CaASMT gene family in pepper plants. Phylogenetic tree of the CaASMT genes was constructed using MEGA7.0, and the CaASMT genes were divided into two, groups I and II.

**Figure 2:**
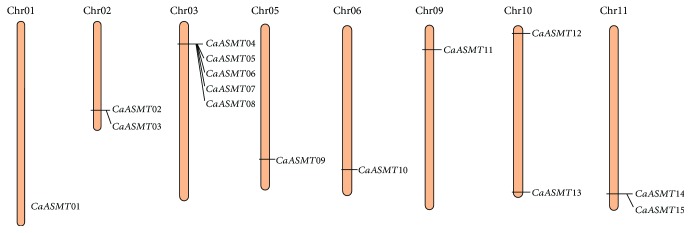
Chromosomal location and tandem duplication of CaASMT genes. Black boxes showed the tandem duplication genes on chromosomes. The chromosome numbers are indicated at the top of chromosomes.

An unrooted phylogenetic tree of 15 CaASMT genes from the pepper species was constructed using MEGA7.0 software based on their protein sequences. CaASMT11 was eliminated due to its large sequence diversity. As displayed schematically in Figure 1, all these CaASMT sequences could be separated into three main groups (group I, II, and III) which are strongly supported by the statistically high bootstrapping values. Nine out of fifteen genes, accounting for 60% in total, were grouped together in group I. Only two members (CaASMT02 and 03) were assigned to group II, (Figures 1 and 2). Within group III, four genes were identified (Figure 1).

**Figure 3:**
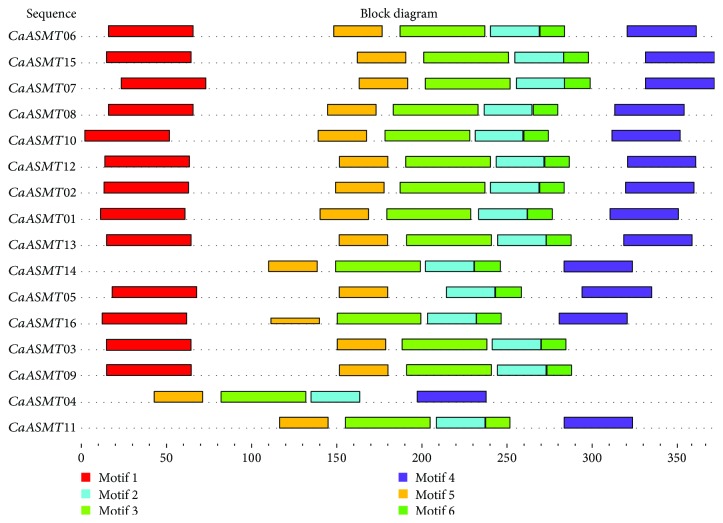
Conserved motif analysis of CaASMT proteins. The characteristic of the putative motifs in CaASMT proteins. Different color boxes represent various types of conserved motifs.

To better understand the diversity of CaASMT proteins, amino acid sequences of 16 genes were analyzed using the online MEME tool (http://meme-suite.org/tools/meme). The result showed 6 distinct motifs (motif 1 to motif 6) (Figure 3). Protein sequences of these motifs varied from 15 to 50 aa in length (*P* < 0.0001) (Table 2). Figure 3 showed that a majority of genes harbor all six motifs, indicating that these proteins were highly conserved during the evolution of pepper plants. However, we found that motif 4 existed in group I and lacked in two genes (CaASMT03, 09) from group II and group III, respectively. The CaASMT14 and CaASMT11 lacked motif 1, and CaASMT04 from group I lacked motif 1 and motif 6.

### 3.3. Chromosome Distribution of the CaASMT Gene Family Members

In this study, chromosome distribution of CaASMT genes was performed based on the pepper genome sequences. The result showed that, among sixteen identified CaASMT members, only fifteen were unevenly distributed on eight of the twelve pepper chromosomes (Figure 2). One of the genes (CaASMT16) was eliminated because it was located on a chromosome 00 (Chr00). None of CaASMT genes was mapped on Chr04, 07, 08, and 12. All CaASMT genes were found in the distal ends of the pepper chromosomes. Each of four chromosomes (Chr01, 05, 06, and 09) only harbor one CaASMT gene. Two genes are found to be present on each of the three chromosomes (Chr02, 10, and 11). The Chr03 have the maximum gene number with five CaASMT genes, accounting for approximately 33% (5/16). However, the sequenced size of Chr03 (261.5 Mb) only accounts for about 7% of the whole pepper genome (3.5 Gb). Subsequently, we further analyzed the tandem and segmental duplications of CaASMT genes in pepper. We found that both of the tandem duplication and segmental duplication events were not found in the CaASMT gene family.

### 3.4. Expression Analysis of CaASMT Genes in Different Tissues

**Figure 4:**
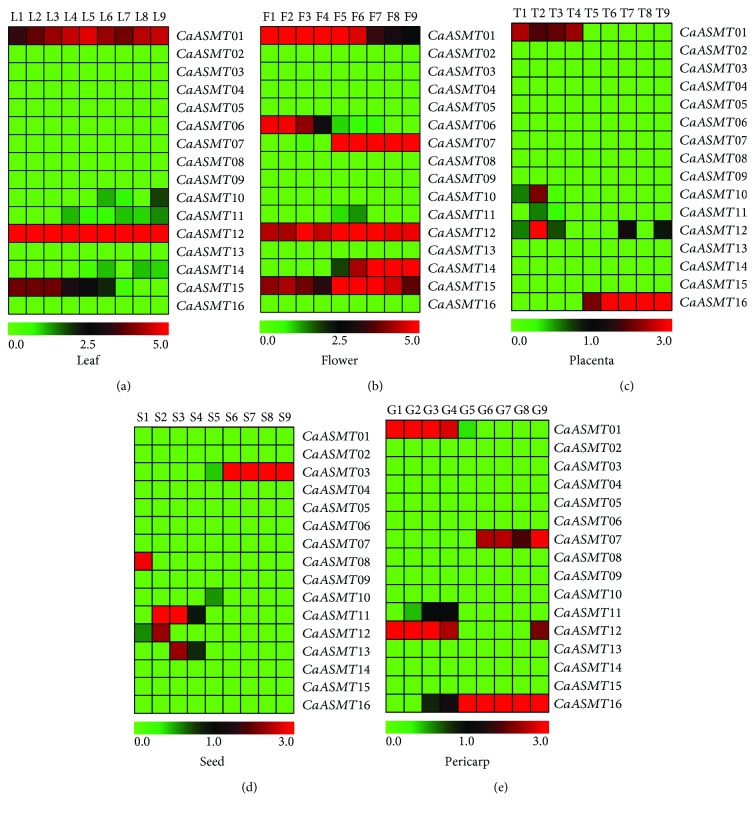
Heat map representation of organ-specific CaASMT gene expression profiles in different tissues of pepper, including leaf (a), flower (b), pericarp (c), seed (d), and placenta (e). The data were normalized based on the mean expression value of each gene in all identified tissues. Then, the log2-transformed RPKM values were used to obtain heat maps using the software MeV 4.9.0. Red and green boxes indicate high and low expression levels of the CaASMT genes, respectively. Leaf samples were collected at 5, 10, 15, 20, 25, 30, 40, 50, and 60 days after emergence (DAE) and were named L1 to L9, respectively. Floral buds were collected at 0.25 cm, 0.35 cm, 0.5 cm, 0.7 cm, 0.8 cm, 1 cm, 1.2 cm, 1.45 cm, and 1.7 cm and were named F1 to F9, respectively. Placenta, seed, and pericarp grown from the tagged flowers were collected at 20, 25, 30, 35, 40, 45, 50, 55, and 60 days after flowering (DAF) and were named T1 to T9, S1 to S9, and G1 to G9, respectively.

It is well known that RNA-seq data has a wide variety of applications, for conveniently assaying the differential expression of genes in higher plants [42]. In the present paper, the RNA-seq data of the three organs from leaf, flower, and fruit were used to analyze the expression patterns of the 16 CaASMT genes. The results revealed that most of CaASMT genes in these three organs were completely unexpressed in all of the examined tissues (Figure 4). In the different development stages of leaf (L1 to L9), the expression levels of ten out of sixteen CaASMT genes were undetectable, including CaASMT02, 03, 04, 05, 06, 07, 08, 09, 13, and 16 (Figure 4(a)). Two genes (CaASMT01 and CaASMT12) were expressed constitutively in all of the stages analyzed. The expression of CaASMT15 is weak and gradually decreases along the development.

The expression patterns of all CaASMT genes in the flower organs were explored, including nine different tested stages (F1-F9). As shown in Figure 4(b), among 16 CaASMT genes, two members (CaASMT12 and 15) were expressed in all nine tested stages. Two genes (CaASMT07 and CaASMT14) showed similar expression patterns, which were found in the investigated stages from F5 to F9. The CaASMT06 was expressed in the previous stages (F1 to F4), and high expression levels of the CaASMT01 gene were observed in F1 to F6 and then downregulated at the late stages analyzed (F7 to F9).

In pepper fruit, three tissues (placenta, seeds, and pericarp) were selected for analyzing the expression patterns of the CaASMT genes. Nine developmental stages in each of the tissues (placenta: T1-T9, seed: S1-S9, and pericarp: G1-G9) were included. In placenta, the result showed that overwhelming majority of the CaASMT genes were at almost undetectable or weakly expressed in the different stages (T1-T9) (Figure 4(c)). The transcripts of the CaASMT16 and CaASMT01 were expressed at the late of stage (T5-T9) and at the early of stage (T1-T4), respectively. The gene CaASMT12 was higher expressed in T2, T7, and T9, and CaASMT10 was in T2.

For seed, five genes (CaASMT03, 08, 11, 12, and 13) were expressed in at least one stage (Figure 4(d)). The CaASMT03 was found highly transcripted in S6-S9. The transcript levels of CaASMT08 were highly expressed in S1. The expression of CaASMT13 and CaASMT12 was observed in S3 to S4 and S2, respectively. The gene CaASMT11 was preferentially expressed in the stage of S2 to S4. In pericarp, the expression of the five members (CaASMT01, 07, 11, 12, and 16) was observed (Figure 4(e)). The CaASMT01 and CaASMT12 genes shared similar expression profiles in several tested stages, such as G1 to G4. The expression of the CaASMT07 was found in G6 to G9. The transcripts of CaASMT11 were observed in G3 and G4, whereas the CaASMT16 was highly expressed from G3 to G9.

### 3.5. Expression of CaASMT Members under Abiotic Stresses

**Figure 5:**
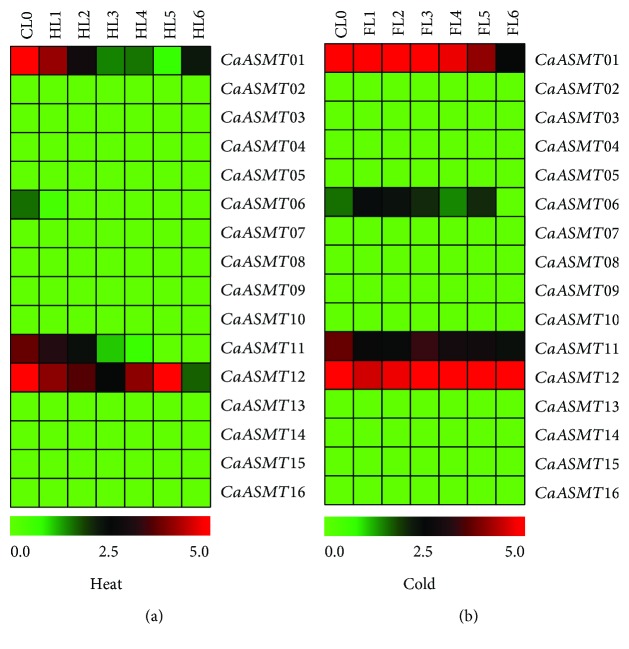
Expression profiles of CaASMT genes in response to abiotic stresses based on RNA-Seq in pepper. (a) Leaf under heat treatment conditions; (b) leaf under cold treatment conditions. Leaf tissue was collected from both heat- and cold-treated plants at 0, 0.5, 1, 3, 6, 12, and 24 hours posttreatment (HPT) and was named HL1 to HL6 and FL1 to FL6, respectively. CL0 represents the control group. All RNA-Seq datasets were obtained from the Pepper Informatics Hub (http://pepperhub.hzau.edu.cn/), and a detailed description of the samples is available from the Pepper Informatics Hub. The data were normalized based on the mean expression value of each gene in all identified tissues. Then, the log2-transformed RPKM values were used to obtain heat maps using the software MeV 4.9.0. Red and green boxes indicate the high and low expression levels of the CaASMT genes, respectively.

To further analyze the expression patterns of CaASMTs in response to stress-related stimuli, the expression profiles of the CaASMT genes in leaf were analyzed under heat and cold conditions (Figure 5). The results revealed that three genes (CaASMT01, 11, and 12) were induced in different levels; the remaining thirteen CaASMT genes were not regulated under heat and cold conditions. For heat stress, two genes (CaASMT01 and 11) share similar expression pattern and were downregulated in HL1 to HL6. The expression levels of the gene (CaASMT12) were irregularly downregulated in all stages (Figure 5(a)). Under cold treatment condition, the CaASMT06 slightly upregulated the stage of FL1 to FL5, whereas the CaASMT11 gene was downregulated in all the stages analyzed (Figure 5(b)). The expression level of CaASMT01 and CaASMT12 was constant in all the stages with the exception that CaASMST01 was downregulated in FL6.

### 3.6. RT-qPCR Analysis

**Figure 6:**
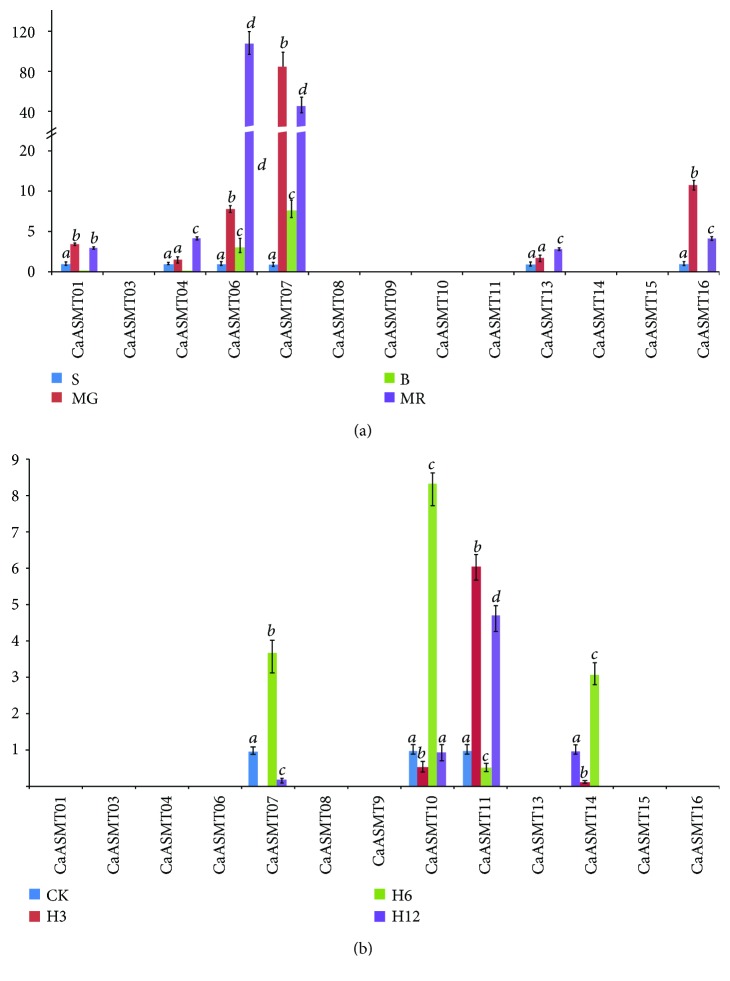
Expression profiles of CaASMT genes in different pepper tissues based on RT-qPCR. (a) Expression profiles of CaASMT genes in fruit. The tested tissues: small fruit (S), mature green fruit (MG), breaker fruit (b), and mature red fruit (MR). (b) Expression profiles of CaASMT genes under heat stress.

To investigate the transcriptional levels of the CaASMT gene family of members, RT-qPCR was used to analyze their expression patterns in different stages of fruit development (small fruit (S), mature green fruit (MG), breaker fruit (B), and mature red fruit (MR)) and under heat stress (Figure 6). The primer pairs of three CaASMT genes (CaASMT02, 05, and 12) can be designed since high GC content in their sequences. For pepper fruits, high expression levels of three CaASMT genes (CaASMT06, 07, and 16) were observed in MG, B, and MR (Figure 6(a)). Three genes (CaASMT01, 04, and 13) were expressed in MR. Under heat stress condition, the transcript levels of four genes (CaASMT07, 10, 11, and 14) were upregulated markedly (Figure 6(b)). In contrast, none of the remaining genes was induced under heat stress condition.

## 4. Discussion

In 2011, Kang et al. reported that the four critical enzymes (TDC, T5H, SNAT, and ASMT) can synthesize melatonin in plant species [16, 17]. Among them, the ASMT is the last enzyme of melatonin biosynthesis and contained three members, while the T5H and SNAT genes encode single copy genes [15, 16]. In the current study, the ASMT gene family was identified using the bioinformatics methods in the *C. annuum* genome. A total of 33 CaASMT homologous were identified in the pepper genome. Of them, more than half of the candidates (17/33) were eliminated since that homologs are less than 31% amino acid identities when the pepper CaASMT protein sequences were aligned to the rice ASMT sequences [40]. Recently, the whole SlASMT gene family was also identified in the tomato genome and only encoded 14 genes [43].

To identify whether the CaASMT genes underwent expression divergence, the transcriptional levels of each member were spatially and temporally analyzed in pepper. In five different organs, most of the CaASMT genes were completely unexpressed in all of the detected stages. In leaf, the CaASMT01 and CaASMT12 were expressed in all of the detected stages, whereas the CaASMT15 was expressed in L1 to L6. We concluded that they are likely to act as a housekeeping gene of pepper cell under normal growth conditions. In flower, high expression levels of three genes (CaASMT01, CaASMT12, and CaASMT15) were observed in all the stages analyzed (F1 to F9), indicating that these genes were involved in the growth and development of flower. The CaASMT07 and CaASMT14 were transcribed at the late stages (F5 to F9), while CaASMT06 was transcribed at the early stages (F1 to F4). These results showed these genes potentially play diverse functional roles in different development stages.

We further analyzed the expression profiles of these CaASMT genes in placenta, seed, and pericarp (Figures 4(c)–4(e)). In placenta, the CaASMT01 and CaASMT16 genes were expressed at the early (T1 to T4) and late (T5 to T9) stage, respectively. The result indicated that these two genes might involve in the capsaicin metabolism. In seed, four genes (CaASMT08, 11, 12, and 13) were stage-preferential expressed at the early stage (S1 to S4), while the transcript of CaASMT03 was transcribed at the late stage (S6 to S9). The results indicated that these genes are related to seed development and maturation in pepper plants. In pericarp, two genes (CaASMT01 and CaASMT12) were expressed at the early stage (G1 to G4), and the CaASMT07 and CaASMT16 were expressed at the late stage (G5 to G9). We concluded that these genes play important roles in the formation and maturity of pepper pericarp. In conclusion, these CaASMT genes may have specific functions at different organs and stages. Our study showed that functional divergence of the CaASMT proteins has occurred during the process of its evolution.

Under abiotic conditions, several CaASMT genes in leaf were induced when plant growth are affected by heat and cold (Figure 5). Under heat stress, the three genes (CaASMT01, 11, and 12) are downregulated in different stages. Under cold stress, three CaASMT genes (CaASMT01 and 09) were slightly induced in the stages tested. Overall, the result showed that three CaASMT genes have changed under heat and cold treatments, suggesting that these CaASMT genes in pepper could be involved in plant response to abiotic stresses. In conclusion, the RNA-seq analysis of the CaASMT gene family provides us a firm foundation for comprehensively understanding the function of ASMT genes in the growth and development of pepper plants, as well as under abiotic stresses. However, the functions of these genes in pepper genome are still unclear, which needs further experimental verification in the future.

The qRT-PCR is widely used for the quantification of the mRNA levels in different tissues, which plays an important role in mRNA expression analysis [44]. The qRT-PCR analysis results showed that 6 out of 13 CaASMT genes were expressed in MG, B, and MR, indicating that these genes might play an important role in the process of fruit maturing. Under heat stress treatment, the expression of CaASMT01 was not detected using qRT-PCR technology. The expression of the two genes (CaASMT6 and 07) was detected, while two of the genes were unexpressed in RNA-seq experiment. These results were different from the results obtained by RNA-seq since the different accessions were used in two experiments.

## Figures and Tables

**Table 1:** The CaASMT genes in pepper.

Gene Name	Locus Name	Chromosome Location	ORF (bp)	Length(aa)^a^	Mw^b^	PI^c^	Index
CaASMT01	Capana01g003955	Chr01:272050432:272052734	1056	351	39.48	5.47	40.4
CaASMT02	Capana02g001874	Chr02:135127565:135129118	1083	360	40.42	5.32	32.24
CaASMT03	Capana02g001870	Chr02:135102434:135103724	1029	342	38.3	5.82	29.68
CaASMT04	Capana03g001635	Chr03:31151757:31152665	717	238	26.22	4.75	30.67
CaASMT05	Capana03g001638	Chr03:31174680:31175943	1026	341	38.86	5.84	38.83
CaASMT06	Capana03g001641	Chr03:31272133:31273344	1086	361	40.31	5.45	37.57
CaASMT07	Capana03g001645	Chr03:31408346:31409581	1119	372	41.81	5.54	34.05
CaASMT08	Capana03g001649	Chr03:32076926:32078500	1065	354	39.08	5.12	29.08
CaASMT09	Capana05g001855	Chr05:178284197:178285864	948	315	35.65	4.83	30.15
CaASMT10	Capana06g002632	Chr06:187528360:187529572	1101	366	41.29	5.72	32.97
CaASMT11	Capana09g000652	Chr09:30401455:30408782	978	325	36.41	6.22	45.27
CaASMT12	Capana10g000326	Chr10:7678051:7680186	1086	361	40.6	5.59	35.8
CaASMT13	Capana10g002309	Chr10:202365592:202367744	1080	359	40.91	5.24	36.16
CaASMT14	Capana11g001924	Chr11:202292166:202293232	975	324	36.9	5.37	34.14
CaASMT15	Capana11g001925	Chr11:202403861:202405079	1119	372	42.04	5.17	36.9
CaASMT16	Capana00g001238	Chr00:365586712:365591893	966	321	36.1	5.55	47.94

^a^Length of the deduced amino acid; ^b^Molecular weight; ^c^Isoelectric point.

**Table 2:** Conserved motifs of CaASMT genes in pepper.

Motif	Width	Sequence
1	50	AAQAHIWNHIFNFINSMSLKCAIQLGIPDIIHSHGKPMTLSDLVDALPIN
2	29	VGGDMFKSIPSADAILLKWILHDWSDEDC
3	50	KDVFEGLKSLVDVGGGTGTVAKAIAKAFPQIKCTVLDLPHVIEGLEGSKN
4	41	VLVSGKERSZQEWAKLFSDAGFSDYKIIPILGLRSIIEVYP
5	29	AHGKPFFEYAGNEPRLNHLFNEAMASDSR
6	15	VKILKKCKEAIPSKE

## Data Availability

The data used to support the findings of this study are available from the corresponding author upon request.

## Supplementary Materials

Supplementary Materials Table S1: primers used to detect the expression of pepper CaASMT genes in this study.
