# Supplementary material for: Analysis of the ASMT Gene Family in Pepper (Capsicum annuum L.): Identification, Phylogeny, and Expression Profiles
Source: Int J Genomics. 2019 Apr 2;2019:7241096. doi: 10.1155/2019/7241096 (PMC6466892; doi:10.1155/2019/7241096)
Supplement: Supplementary Materials — Table S1: primers used to detect the expression of pepper CaASMT genes in this study. [file 7241096.f1.doc]

Table S1: Primers used to detect the expression of pepper CaASMT genes in this study

| Name | Locus gene | Forward primer (5’ to 3’) | Reverse primer (5’ to 3’) |
| --- | --- | --- | --- |
| CaAMST01 | Capana03g001649 | GGATCCATGGCAATCACTCG | AACACACTCGTGATCAACCG |
| CaAMST02 | Capana09g000652 | GATGTAGGTGGAGGGATAGGG | CCAATCATGCATCACCCACT |
| CaAMST03 | Capana03g001645 | ACTGGAACCGTGGCTAAGTT | AGGTTCTTGCTCCCTTCACA |
| CaAMST04 | Capana11g001925 | ACAGAAGAGGAGGAGGAGGA | TCTAGTCCCACCTGCACAAG |
| CaAMST06 | Capana03g001641 | GGCTCAAGAATGGTGACGAC | AATGGTTAAGCCACGGTTCG |
| CaAMST08 | Capana11g001924 | CATTGGTGGACGTAGGAGGT | GTGGCAGATCGAAGACGATG |
| CaAMST09 | Capana01g003955 | GGCTAGTGAGTCGAGGTTGA | CCCAGTACTGCTCTTGAGGT |
| CaAMST10 | Capana06g002632 | ATCCAATTTGGTGGCTGCTC | ACGTGAAGCCGGAGTAAGTA |
| CaAMST11 | Capana10g002309 | GCCTTGGAACTCGATGGAAG | TCGCGAATCTCTAGCCATGT |
| CaAMST12 | Capana00g001238 | GTCGTTGGTGGATGTTGGAG | AGGTCGCCTATCACATGAGG |
| CaAMST13 | Capana02g001870 | TGGTGCACCCAGGATTCTTT | TTGCCAAGGAGTGACAAAGC |
| CaAMST14 | Capana03g001635 | TGAAGGATGTGAAGGGAGCA | TTGCATCGGCAGAAGGAATG |
| CaAMST15 | Capana05g001855 | GCCTTGGAACTCGATGGAAG | TTCGCGTGAAACCAGTTACC |
